# The DAS28-ESR cutoff value necessary to achieve remission under the new Boolean-based remission criteria in patients receiving tocilizumab

**DOI:** 10.1007/s10067-012-2103-4

**Published:** 2012-10-23

**Authors:** Yasuhiko Hirabayashi, Tomonori Ishii, for the Michinoku Tocilizumab Study Group

**Affiliations:** 1 Department of Rheumatology, Hikarigaoka Spellman Hospital, 6-7-1 Higashisendai, Miyagino-ku, Sendai, 983-0833 Japan; 2 Department of Hematology and Rheumatology, Tohoku University Hospital, Sendai, Japan

**Keywords:** Boolean, Criteria, DAS28-ESR, Remission, Tocilizumab

## Abstract

To seek the cutoff value of the 28-joint disease activity score using erythrocyte sedimentation rate (DAS28-ESR) that is necessary to achieve remission under the new Boolean-based criteria, we analyzed the data for 285 patients with rheumatoid arthritis registered between May 2008 and November 2009 by the Michinoku Tocilizumab Study Group and observed for 1 year after receiving tocilizumab (TCZ) in real clinical practice. Remission rates under the DAS28-ESR criteria and the Boolean criteria were assessed every 6 months after the first TCZ dose. The DAS28-ESR cutoff value necessary to achieve remission under the new criteria was analyzed by receiver operating characteristic (ROC) analysis. Data were analyzed using last observation carried forward. After 12 months of TCZ use, remission was achieved in 164 patients (57.5 %) by DAS28-ESR and 71 patients (24.9 %) under the new criteria for clinical trials. CRP levels scarcely affected remission rates, and the difference between remission rates defined by DAS28-ESR and by the new criteria was mainly due to patient global assessment (PGA). Improvement of PGA was inversely related to disease duration. ROC analysis revealed that the DAS28-ESR cutoff value necessary to predict remission under the new criteria for clinical trials was 1.54, with a sensitivity of 88.7 %, specificity of 85.5 %, positive predictive value of 67.0 %, and negative predictive value of 95.8 %. A DAS28-ESR cutoff value of 1.54 may be reasonable to predict achievement of remission under the new Boolean-based criteria for clinical trials in patients receiving TCZ.

## Introduction

Rheumatoid arthritis (RA) is a chronic inflammatory disease which fluctuates in activity during the course of the disease. To evaluate disease activity at the time of observation, the 28-joint disease activity score using erythrocyte sedimentation rate (DAS28-ESR) was developed in 1995 and the cutoff value representing remission was defined as <2.6 [1]. However, we now know that multiple joints can remain swollen or tender at this cutoff value. Because several biologics have now become available, achievement of complete clinical remission has become a practical goal and the DAS28-ESR definition of remission has now become unsuited for use in real clinical practice. Therefore, a new definition of remission based on a Boolean approach was approved by the American College of Rheumatology (ACR) and the European League Against Rheumatism (EULAR) [2]. The new criteria for clinical trials define remission to be when tender joint count (TJC), swollen joint count (SJC), CRP (in milligrams per deciliter), and scores on a patient global assessment using a visual analogue scale (PGA-VAS) (0–10-cm scale) are all ≤1. The same criteria, but excluding CRP, are the new criteria for defining remission in clinical practice.

DAS28-ESR has been used worldwide and contributes considerably to the standardization of evaluation of disease activity. If the DAS28-ESR cutoff value necessary to achieve remission under the new ACR/EULAR Boolean-based criteria is found, it will be possible to reanalyze a lot of the accumulated data based on DAS28-ESR. In this report, we sought this cutoff value on the basis of prospectively registered observational data by the Michinoku Tocilizumab Study Group (MTSG) which was organized to evaluate the efficacy and safety of tocilizumab (TCZ) in real clinical practice.

## Subjects and methods

### Subjects

The subjects of this analysis were patients meeting the 1987 revised RA classification criteria from the ACR and who had newly received TCZ following its marketing approval in Japan for use in RA. A total of 285 patients in 34 institutions in the Tohoku area were registered between May 2008 and November 2009 by the MTSG. Their demographics and baseline characteristics prior to treatment are as follows: mean age ± standard deviation (SD) was 59.6 ± 13.1 years; male/female ratio was 21:79; mean disease duration ± SD was 10.2 ± 9.1 years; the percentage of patients of Steinbrocker’s classes I, II, III, and IV was 21, 57, 22, and 0 %, respectively; Steinbrocker’s stages I, II, III, and IV was 10, 23, 29, and 38 %, respectively; mean TJC ± SD was 6.5 ± 6.0; mean SJC ± SD was 5.6 ± 5.1; mean ESR ± SD was 50.6 ± 32.8 mm/h; mean CRP ± SD was 2.7 ± 2.8 mg/dl; mean PGA-VAS ± SD was 5.43 ± 2.47 cm (on a 0–10-cm scale); and mean DAS28-ESR ± SD was 5.2 ± 1.3.

### Methods

This study was not an intervention study and was approved by the Ethics Committee of Tohoku University. The patients had been observed for 1 year after receiving TCZ in real clinical practice. TJC, SJC, PGA-VAS, ESR, and CRP were assessed every 6 months after the first TCZ dose to calculate the remission rates under the DAS28-ESR definition and under the new 2011 ACR/EULAR Boolean-based remission criteria. The DAS28-ESR cutoff value necessary to achieve remission under the new criteria was analyzed by receiver operating characteristic (ROC) analysis. Data were analyzed using last observation carried forward.

## Results

### Proportions of patients achieving the criteria or each component of the criteria

**Fig. 1:**
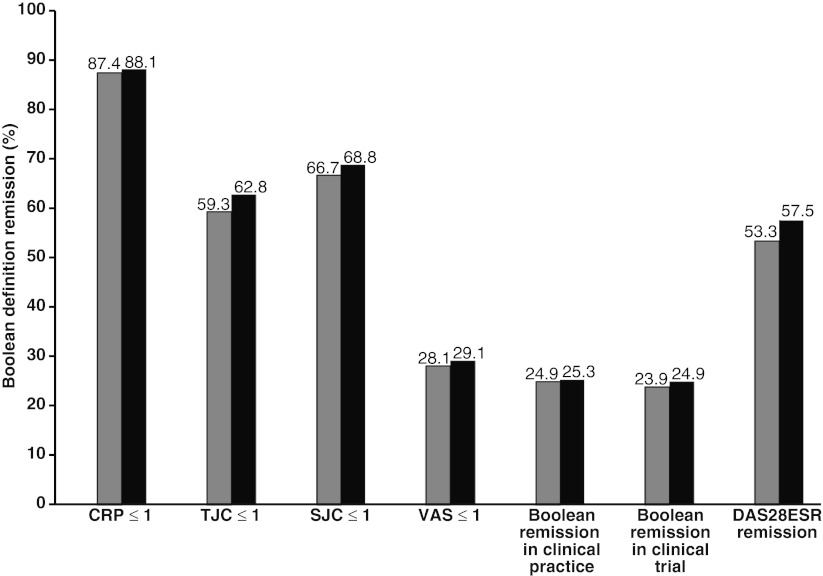
Percentage of patients achieving each component of the Boolean-based remission criteria or achieving remission at 6 months (*gray bars*) or 12 months (*black bars*)

In the 285 patients, the 6-month- and 12-month TCZ continuation rates were 87.4 and 81.8 %, respectively. After 6 months of TCZ use, remission was achieved by 152 patients (53.3 %) under the DAS28-ESR definition, by 71 patients (24.9 %) under the new criteria for clinical practice, and by 68 patients (23.9 %) under the new criteria for clinical trials (Fig. 1). After 12 months, remission was achieved by 164 patients (57.5 %) under the DAS28-ESR definition, by 72 patients (25.3 %) under the new criteria for clinical practice, and by 71 patients (24.9 %) under the new criteria for clinical trials. ESR decreased from 50.6 ± 32.8 mm/h at baseline to 14.2 ± 21.8 after 6 months and to 13.9 ± 21.7 after 12 months. The mean DAS28-ESR decreased from 5.2 at baseline to 2.7 after 6 months and to 2.6 after 12 months. Next, the number of patients achieving each component of the Boolean-based remission criteria was tallied. CRP ≤1 mg/dl was achieved by 249 patients (87.4 %) at 6 months and 251 patients (88.1 %) at 12 months. TJC ≤1 was achieved by 169 patients (59.3 %) at 6 months and 179 patients (62.8 %) at 12 months. SJC ≤1 was achieved by 190 patients (66.7 %) at 6 months and 196 patients (68.8 %) at 12 months. PGA-VAS ≤1 cm was achieved by 80 patients (28.1 %) at 6 months and 83 patients (29.1 %) at 12 months.

### DAS28-ESR cutoff value necessary to predict achievement of remission under the new criteria

**Fig. 2:**
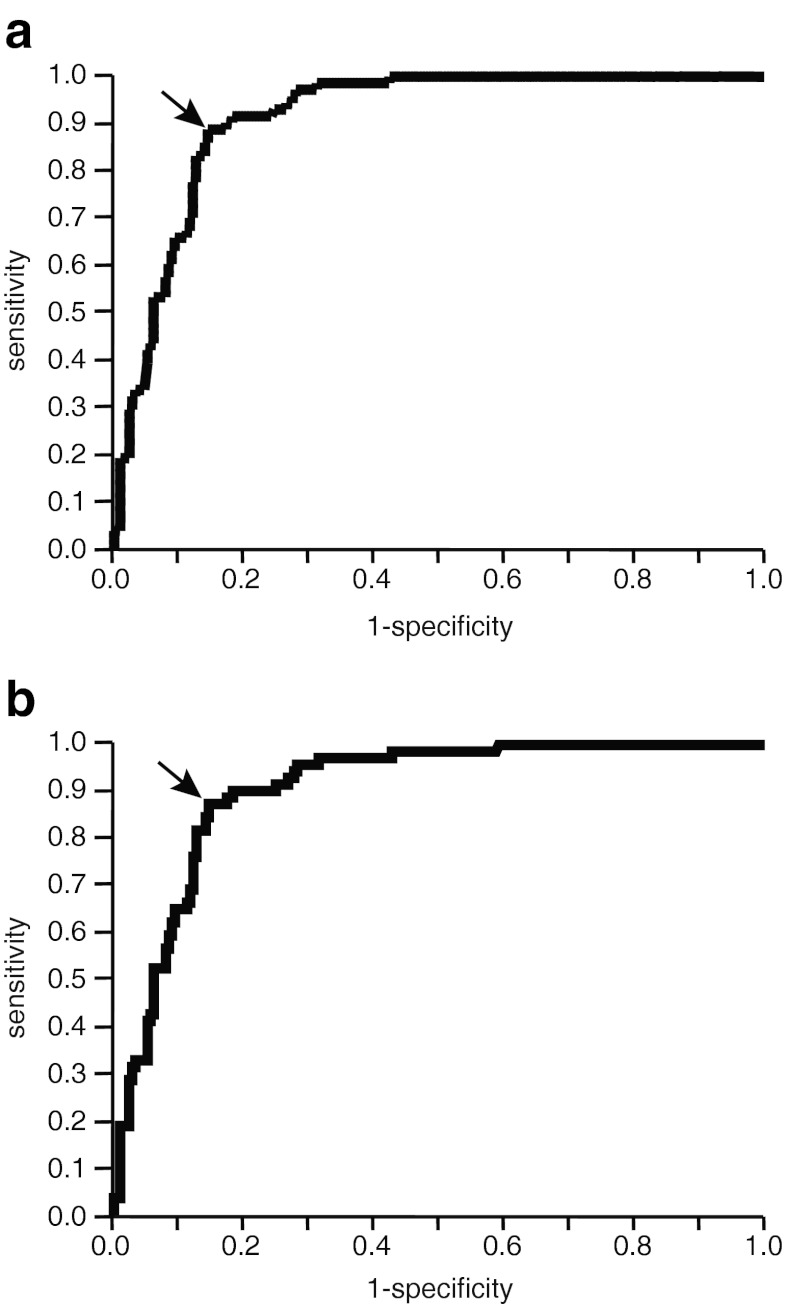
Receiver operating characteristic curve of DAS28-ESR <1.54 for the ACR/EULAR Boolean-based definition of remission in clinical trials (**a**) and in clinical practice (**b**). The *arrow* indicates the point where the value of [sensitivity − (1 − specificity)] becomes maximum

The DAS28-ESR cutoff values necessary to achieve remission under the new criteria for clinical trials and for clinical practice were analyzed by ROC analysis (Fig. 2). The analysis revealed that a value of 1.54 was the most accurate cutoff value for both sets of criteria, with a sensitivity of 88.7 % (trials) and 87.5 % (practice), specificity of 85.5 % (trials) and 85.5 % (practice), positive predictive value of 67.0 % (trials) and 67.0 % (practice), and negative predictive value of 95.8 % (trials) and 95.3 % (practice). The area under the ROC curve was 0.91 in both sets of criteria, indicating high accuracy.

### Effect of disease duration on achievement of remission

**Fig. 3:**
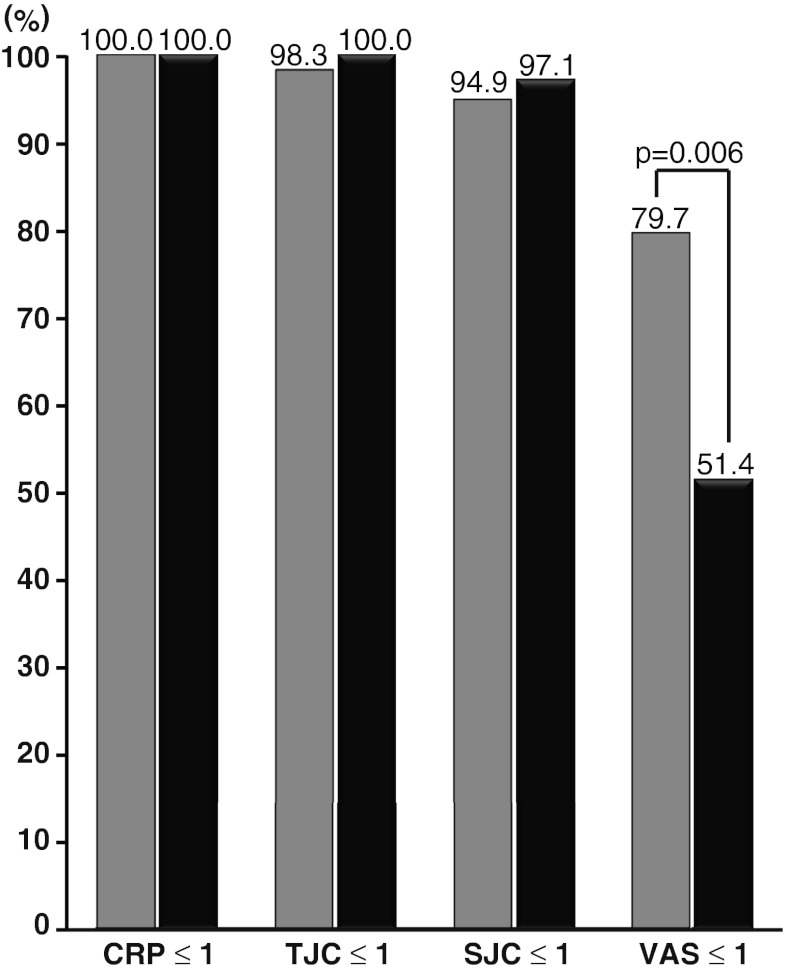
Percentages of patients with DAS28-ESR <1.54 achieving each component of the Boolean-based remission criteria compared by disease duration. *Gray bars* disease duration <10 years, *black bars* disease duration ≥10 years

Compared to the negative predictive value, the positive predictive value was not so high. To clarify the reason for this, we compared the proportions of patients with DAS28-ESR <1.54 achieving each component of the remission criteria in the following two groups: those with disease duration <10 years (Group 1; *n* = 59) and those with disease duration ≥10 years (group 2; *n* = 35) (Fig. 3). CRP ≤1 mg/dl was achieved by 59 patients (100.0 %) in group 1 and 35 patients (100.0 %) in group 2. TJC ≤1 was achieved by 58 patients (98.3 %) in group 1 and 35 patients (100.0 %) in group 2. SJC ≤1 was achieved by 56 patients (94.9 %) in group 1 and 34 patients (97.1 %) in group 2. In these three components, there was no significant difference between the two groups. On the other hand, a significant difference was observed in PGA-VAS. PGA-VAS ≤1 cm was achieved by 47 patients (79.7 %) in group 1 but only 18 patients (51.4 %) in group 2. Thus, PGA-VAS is the key component which determines the remission rate, and the improvement of PGA-VAS is inversely related to disease duration.

## Discussion

In this study, our results demonstrated a high rate of DAS28-ESR remission (53.3 %) after 6 months, which is compatible with the results of our previous study [3]. Even under the new criteria, about 24 % of patients achieved remission. The continuation rates at 6 and 12 months were also high. SJC <1 was achieved by 67–69 % of patients indicating that inflammation had mostly subsided. These results are comparable to those of the phase IIIb TAMARA study [4]. There was no significant difference between the remission rate as determined by the new criteria for clinical trials and that for clinical practice (*P* = 0.923). This means that, although TCZ stops CRP gene transcription [5], the remission rate was scarcely affected by CRP. The discrepancy between DAS28-ESR and the new criteria was mainly due to PGA (Fig. 1).

ROC analysis revealed that the DAS28-ESR cutoff point necessary to predict achievement of remission under the new criteria, both for clinical trials and for clinical practice, was DAS28-ESR <1.54 (Fig. 2). This also means that this cutoff value is not affected by CRP. Sensitivity and specificity were reasonably high. Negative predictive value was very high (>95 %) and the area under the ROC curve was >0.9, indicating high accuracy. Moreover, the cutoff value is clearly below the more stringent DAS28-ESR cutoff point (DAS28-ESR <2.0) examined by the ACR and the EULAR as a candidate definition of remission [2]. Therefore, DAS28-ESR <1.54 may be a reasonable cutoff value to predict achievement of remission under the new ACR/EULAR Boolean-based criteria for TCZ. Of course, it is not known whether this value can be used for other antirheumatic drugs until other drugs are studied in the same way because TCZ strongly improves ESR by markedly ameliorating inflammatory anemia [6] and decreasing inflammatory molecules such as fibrinogen [7].

A new definition of remission based on the simplified disease activity index (SDAI) was also approved by the ACR and the EULAR [2]. However, in this study, we could not indicate a DAS28-ESR cutoff value predicting SDAI <3.3 because the prospectively registered observational data did not include the physician global assessment. We would like to investigate this when we have a chance.

In general, longer disease duration is associated with more severe joint destruction. Understandably, joint destruction decreases quality of life and increases the points on the Health Assessment Questionnaire (HAQ). This increased HAQ score is the damage-related component of the HAQ (DAM-HAQ) of which improvement is not easy [8, 9]. At the damaged joints, motion pain frequently remains even after treatment has resulted in disappearance of inflammation. Therefore, although most patients achieved TJC ≤1 and SJC ≤1, improvement of PGA-VAS is limited in the patients with longer disease duration (Fig. 3). This means that, due to DAM-HAQ, criteria using PGA-VAS could be inaccurate for evaluating disease activity in patients with longer disease duration. One way to improve the accuracy of assessing remission would be to change the cutoff value of PGA-VAS depending on the disease duration or the degree of joint destruction such as determined by the modified total Sharp score.
